# Editorial: Hot trends in computer-aided drug design techniques

**DOI:** 10.3389/fcimb.2023.1149994

**Published:** 2023-04-17

**Authors:** Luciana Scotti, Jagdish Suresh Patel, Malihe Hassanzadeh, Marcus T. Scotti

**Affiliations:** ^1^ Postgraduate Program in Natural and Synthetic Bioactive Products, Federal University of Paraíba, João Pessoa, Brazil; ^2^ University Hospital, Federal University of Paraíba, João Pessoa, Brazil; ^3^ Department of Chemical & Biological Engineering, Moscow, United States; ^4^ Department of Pharmacology and Physiology in Université de Sherbrooke, QC, Sherbrooke, Canada

**Keywords:** CADD, *in silico*, computer-aided drug design, drug research, molecular properties

The drug discovery process is complex, and designing an effective and commercially viable drug requires interdisciplinary work. For this reason, the Computer Aided Drug Design (CADD) Centre works in collaboration with structure biologists, biophysicists, and computational scientists to find new therapeutic agents. The design and development of any medicine takes many years: it begins when scientists learn about a biological target (e.g., a receptor, enzyme, protein, gene) that is involved in a biological process thought to be dysfunctional in patients with a disease, followed by the determination of a specific target receptor and often by the determination of an active compound from the mass of compounds. The target could prevent an altered biological process without being dysfunctional itself (de Araujo et al., 2020; de Sousa et al., 2021; de Araujo et al., 2022).

In this collection, we—researchers of *in silico* methods—focused on the publication of papers that take computer-assisted approaches such as:

- Structure-based drug design
- Virtual screening
- Combining docking and molecular dynamics simulations
- Pharmacophore modeling
- Statistical methods (quantum chemistry calculation and 3D quantitative structure–activity relationships (QSAR) methods)
- Ligand-binding pocket prediction
- Pharmacokinetics/pharmacodynamics (PK/PD) prediction
- Physical property prediction
- Homology modeling to identify novel bioactive compounds.

We are glad about our work, because this issue has amassed 10,000 views and five high-quality manuscript submissions.

The review by Oliveira et al., entitled *Biological Membrane-Penetrating Peptides: Computational Prediction and Applications*, discussed some classes of peptides that are able to naturally cross the biological membranes, such as the cell membrane and blood–brain barrier (BBB). Cell-penetrating peptides (CPPs) and blood–brain barrier-penetrating peptides (B3PPs) have been explored by the biotechnological and pharmaceutical industries to develop new therapeutic molecules and carrier systems. The computational prediction of peptides’ penetration into biological membranes has emerged as an interesting strategy due to their high throughput and low-cost screening of large chemical libraries.

Kumar et al. reported their findings in the research paper entitled *3D-QSAR-Based Pharmacophore Modeling, Virtual Screening, and Molecular Dynamics Simulations for the Identification of Spleen Tyrosine Kinase Inhibitors.* The primary goal of this research was to identify potential inhibitors with higher affinity, higher selectivity based on key molecular interactions, and better drug-like properties than the available inhibitor, fostamatinib. In this study, a 3D-QSAR model was built for SYK based on known inhibitor IC_50_ values. The best pharmacophore model was then used as a 3D query to screen a drug-like database to retrieve hits with novel chemical scaffolds. The obtained compounds were subjected to binding affinity prediction using the molecular docking approach, and the results were subsequently validated using molecular dynamics (MD) simulations.

*In silico investigation and potential therapeutic approaches of natural products for COVID-19: Computer-aided drug design perspective* is the review by Rahman et al. Through a computational approach, the study contributed to the development of effective treatment methods by examining the mechanisms related to the binding and subsequent inhibition of the SARS-CoV-2 ribonucleic acid (RNA)-dependent RNA polymerase (RdRp). The *in silico* method was also employed to determine the most effective drug on the mentioned compound, and their aquatic, non-aquatic, and pharmacokinetic data were analyzed.

Drug development is a lengthy and risky work that requires significant money, resources, and labor. Breast and lung cancer contribute to the death of millions of people throughout the world each year, according to the report by the World Health Organization, and has been a public threat worldwide, although the global medical sector is developed and updated day by day. However, no proper treatment has been found until now. Therefore, research has been conducted to find a new bioactive molecule to treat breast and lung cancer—such as natural myricetin and its derivatives—by using the latest and most authentic computer-aided drug design approaches. Drug-likeness, ADME, and toxicity prediction were fulfilled in the investigation of Akash et al. titled *Development of new bioactive molecules to treat breast and lung cancer with natural myricetin and its derivatives: A computational and SAR approach.* It was noted that all the derivatives were highly soluble in a water medium, whereas they were totally free from AMES toxicity, hepatotoxicity, and skin sensitization, excluding only two ligands. Thus, the authors proposed that the natural myricetin derivatives could be a better inhibitor for treating breast and lung cancer.

*Lianhua Qingwen* granules (LHQW) can reduce tissue damage caused by inflammatory reactions and relieve patients’ clinical symptoms. Cao et al. employed bioinformatics to screen active ingredients in LHQW and intersection gene targets. PPI, GO, and KEGG were used to analyze the relationship between the intersection gene targets. Molecular dynamics simulations validated the binding stability of the active ingredients and target proteins. The binding free energy, radius of gyration, and solvent accessible surface area were analyzed by a supercomputer platform. This study was reported in their research article titled *Molecular docking and molecular dynamics study Lianhua Qingwen granules (LHQW) treats COVID-19 by inhibiting inflammatory response and regulating cell survival.*

We, the guest editors, would like to express our gratitude to the many authors who contributed to this Research Topic, reporting investigations in various aspects of *Hot Trends in Computer-Aided Drug Design Techniques.*

## Author contributions

All authors listed have made a substantial, direct, and intellectual contribution to the work and approved it for publication.
